# The relationship between psychological resilience, decision confidence, and officiating pressure of college basketball referees: a cross-lagged study

**DOI:** 10.3389/fpsyg.2026.1793112

**Published:** 2026-04-15

**Authors:** Zhihao Feng, Zhengyang Zhao, Tong Wang, Qian Luo, Yongfeng Liu

**Affiliations:** School of Sports Training, Chengdu Sport University, Chengdu, Sichuan, China

**Keywords:** college basketball referees, cross-lagged, decision confidence, officiating pressure, psychological resilience

## Abstract

**Objectives:**

To address the limitations of prior cross-sectional studies, this study employed a longitudinal follow-up design to investigate the reciprocal longitudinal relationships among psychological resilience, decision confidence, and officiating pressure.

**Methods:**

Over a 6-month period, 368 college basketball referees were surveyed three times using the Officiating and Decision Stress Scale for Referees (SASS-SO), the 10-item Connor-Davidson Resilience Scale (CD-RISC-10), and the Referee Self-Efficacy Scale (REFS). Longitudinal measurement invariance was assessed using Amos 29.0, and a cross-lagged panel model was employed to examine the relationships among the three variables.

**Results:**

The cross-lagged panel model demonstrated an acceptable fit to the data (*χ*^2^/df = 1.257, CFI = 0.949, TLI = 0.983, RMSEA = 0.026). Psychological resilience was positively associated with subsequent decision confidence and negatively associated with subsequent officiating pressure over time. Decision confidence was negatively associated with subsequent officiating pressure but was not significantly associated with subsequent psychological resilience. Officiating pressure was negatively associated with subsequent psychological resilience and with decision confidence.

**Conclusion:**

Psychological resilience was positively associated with subsequent decision confidence and negatively associated with subsequent officiating pressure over time. Decision confidence was negatively associated with subsequent officiating pressure. Officiating pressure was negatively associated with subsequent psychological resilience and with decision confidence. Decision confidence also mediated the indirect longitudinal relationship between psychological resilience and officiating pressure. These findings should be interpreted as predictive rather than causal, given the limitations of the traditional CLPM.

## 1 Introduction

In basketball, referees are integral to the sport. Every decision they make directly impacts athletes’ technical and tactical performance, as well as the fairness of the game. Therefore, referees are both the “enforcers” of the rules and the “servants” of the game (Sabag et al., 2023). High-level competitions are typically characterized by high intensity and a fast pace, requiring referees to possess excellent psychological qualities and judgment skills. Only by meeting both intense mental and physical demands can referees maintain the accuracy and fairness of officiating (Liu, 2016). As a unique group with dual identities as “students” and “referees,” college basketball referees face on-court officiating that is immediate, complex, and irreversible. Consequently, they often experience excessive psychological pressure, low decision confidence, and poor stress resistance during officiating. The majority of existing studies in this area have used cross-sectional designs, and research on the relationships among these three variables remains limited. To address the limitations of cross-sectional studies, this research employed a longitudinal design using a cross-lagged panel model, recruiting National Class 1 basketball referees from the college student population. During the 6-month study period, these referees were surveyed three times at 3-month intervals to examine the relationships among psychological resilience, decision confidence, and officiating pressure. The article is structured as follows: The first part is a literature review of psychological resilience, decision confidence, and officiating pressure. After a comprehensive review of the relevant literature, we propose the possible relationships among the variables. Then, we proposed the research methods, as well as the measurement of variables and the construction of the cross-lagged panel model. Finally, the research results are reported and discussed, while the existing limitations and future research directions are highlighted.

## 2 Literature review

### 2.1 Psychological resilience

Psychological resilience is defined as the capacity to maintain stable judgments and mental states when confronting competitive pressure and penalty-related disputes (Xi and Sang, 2008; Leicht et al., 2017). The American Psychological Association defines psychological resilience as the process of adapting well in the face of adversity, trauma, tragedy, threats, or significant stressors (Southwick et al., 2014). In referees, psychological resilience has a specific contextual nature. It not only buffers external pressures (e.g., audience interference, player pressure, and public opinion) but also enables consistent judgments and decisive decision-making. It also effectively suppresses anxiety when facing uncertain situations, such as critical calls or challenges from the head coach, allowing referees to maintain a rational assessment of rules and circumstances and make accurate, decisive judgments (Coulter et al., 2010; Hill and Hemmings, 2015). Previous studies have shown that in team sports such as basketball, cognitive training, stress inoculation training, and team support can effectively improve athletes’ psychological resilience (Connaughton et al., 2008), and some research has found that psychological resilience not only negatively predicts athletes’ psychological fatigue but also serves as a chain-mediator in sports behaviors through habit control and resisting temptation (Zheng and Muratli, 2025). Gross and John (2003) further analyzed from the perspective of emotion regulation and proposed that cognitive reappraisal and expressive suppression are the core strategies affecting psychological resilience, among which cognitive reappraisal is more inclined to maintain positive emotional experiences. In addition, psychological resilience is typically manifested when an individual is exposed to pressure or adversity, and psychological resilience is related to decision confidence (Liu et al., 2020). Research indicates that psychological resilience is also a protective factor for decision confidence, and individuals with high psychological resilience often have higher decision confidence (Xi and Sang, 2008), thus maintaining firm judgments in the face of doubts. At the same time, psychological resilience is also a core psychological resource for buffering the negative effects of officiating pressure, which can regulate stress responses and prevent stress from converting into anxiety or professional burnout. Gucciardi et al. (2017) proposed that psychological resilience can not only help athletes cope with competition pressure but also promote recovery after high-pressure situations and the continuous development of their careers.

### 2.2 Decision confidence

Decision confidence refers to referees’ internal sense of certainty when making instantaneous judgments under high-speed, high-pressure, and uncertain game situations, based on limited information and rules (Zhang, 2020b; Samuel et al., 2021). Referee decision confidence and referee self-efficacy are closely related but not identical constructs. Referee self-efficacy generally refers to officials’ beliefs in their capacity to perform successfully in their officiating role. In the referee efficacy framework, decision-making skills are regarded as a core dimension of referee efficacy, together with game knowledge, communication, and coping with pressure. Therefore, in the officiating context, decision confidence may be viewed as a domain-specific component of broader referee self-efficacy (Guillén and Feltz, 2011). Myers et al. (2012) found that referees with high decision confidence are more capable of resisting pressure from players, coaches, and spectators, thereby maintaining the independence and consistency of their judgments. López found that older referees demonstrate higher officiating levels, possess more experience, and exhibit significantly greater decision confidence. Further research revealed that by enhancing theoretical knowledge and on-the-spot experience, decision confidence can be indirectly strengthened. Additionally, there is a close interactive relationship between decision confidence and officiating pressure (Aguilar et al., 2021). Samuel et al. (2021) proposed that referees with high decision confidence tend to adopt proactive problem-solving strategies, actively collecting information, evaluating problems, and making quick decisions, while those with low decision confidence are more likely to fall into avoidance or emotional responses, exacerbating cognitive load and stress perception. Moreover, decision confidence enhances psychological security and control, buffering the negative impact of external stressors on psychological state (Sabag et al., 2023). Gao et al. (2017) reported that there is a mutually reinforcing positive correlation between decision confidence and psychological resilience. Diotaiuti suggested that factors such as referee level, age, and officiating years have a significant impact on the decision information of high-level event referees, so stable decision confidence is an important prerequisite for the psychological aspect of referees during on-the-spot officiating (Diotaiuti et al., 2017). In practice, based on observations of college basketball league officiating, it was found that when college referees are continuously exposed to pressure from coaches and players, their decision confidence is undermined, and they begin to doubt their own judgments.

### 2.3 Officiating pressure

Officiating pressure refers to the dynamic process through which a referee experiences physiological, psychological, and behavioral responses due to interactions with the external environment during officiating (Lazarus and Folkman, 1984; Wang, 2016). Major stressors mainly include psychological burdens regarding the game results and maintaining attention under the fast-paced confrontation, as well as emotional disturbances and verbal provocations from the audience and the team. There are significant differences among groups, with the core stress for university referees coming from insufficient judgment experience and lack of self-confidence (Wang, 2020), and in some regions, referees face unique problems such as language communication barriers, imperfect event organization, and scarce professional training resources (Wu and Yan, 2020). Australian scholar Leicht’s heart rate monitoring research indicates that international-level basketball referees can reach 70–73% HRmax during officiating, approaching the limit of aerobic exercise, and the physiological load and psychological pressure can lead to an imbalance in attention allocation, thereby affecting the speed and accuracy of judgments (Leicht et al., 2017). At the same time, the pressure on high-level referees is also influenced by mood state and psychological resilience. Self-control ability can promote emotional stability through optimizing mood and enhancing psychological resilience to cope with stress (Wang and Yang, 2021). On the other hand, strategies for coping with officiating pressure can be divided into proactive coping and reactive coping. Proactive coping includes skill training, situation rehearsal, and psychological preparation; reactive coping includes emotion regulation (such as deep breathing and mindfulness training), cognitive restructuring, and behavioral adjustment (Aspinwall and Taylor, 1997; Kabat-Zinn, 2003). Mellalieu et al. (2009) emphasized that stress management should be integrated into referee training systems, and its effectiveness can be enhanced through regular assessments and personalized support (Mellalieu et al., 2009). Further research indicates that high-level decision confidence helps referees generate self-confidence and a sense of hope, and a positive sense of hope helps referees develop psychological resilience to resist stress. If referees have good psychological resilience, they can help them actively adapt to adverse situations and stress on the sports field, thereby helping referees improve their individual performance and professional ability and buffer their stress (Locke, 1997; McManama O’Brien et al., 2021).

### 2.4 Theoretical framework and hypotheses

This study was informed by Conservation of Resources (COR) theory and cognitive appraisal theory. According to COR theory, individuals seek to acquire, preserve, and protect valued resources, and stress emerges when these resources are threatened or depleted. In the present study, psychological resilience and decision confidence were viewed as important psychological resources, whereas officiating pressure was understood as a stress state reflecting resource threat and loss.

Cognitive appraisal theory further suggests that stress depends on how individuals evaluate situational demands relative to their coping resources. Referees with greater psychological resilience may be more likely to appraise challenging officiating situations as controllable, thereby experiencing lower subsequent officiating pressure. Similarly, stronger decision confidence may increase perceived control and reduce uncertainty during officiating, which may also alleviate subsequent pressure. In contrast, persistent officiating pressure may gradually consume personal resources and weaken both resilience and confidence over time.

Based on these perspectives, this study proposed that psychological resilience would positively predict subsequent decision confidence and negatively predict subsequent officiating pressure, that decision confidence would negatively predict subsequent officiating pressure, and that officiating pressure would negatively predict subsequent psychological resilience and decision confidence. Accordingly, the following hypotheses were proposed.

*H1*: Psychological resilience will be positively associated with subsequent decision confidence.

*H2*: Psychological resilience will be negatively associated with subsequent officiating pressure.

*H3*: Decision confidence will be negatively associated with subsequent officiating pressure.

*H4*: Officiating pressure will be negatively associated with subsequent psychological resilience and decision confidence.

*H5*: Decision confidence will be involved in the longitudinal indirect association between psychological resilience and officiating pressure.

### 2.5 Cross-lagged panel modeling

Cross-lagged panel modeling (CLPM) is a statistical method based on longitudinal data, used to examine reciprocal temporal associations and dynamic relationships among two or more variables. Its core lies in examining the possible temporal ordering and relative strength between variables by comparing the “lag effects” of the variables at different time points. In psychological research, many studies on the mutual influence between variables have long relied on cross-sectional designs, which makes it difficult to reveal dynamic mechanisms. The cross-lagged panel model provides an effective analytical framework for such longitudinal studies. This model collects measurement data of the same group of subjects at two or more time points and, based on controlling the stability of the variables themselves (autoregressive effect), can examine the cross-time lag relationships between variables, thereby providing a basis for evaluating possible directional temporal associations (Rogosa, 1980; Schuurman et al., 2016). Cross-lagged studies typically evaluate the directional associations between variables by comparing the magnitudes of standardized regression coefficients, which helps analyze the bidirectional longitudinal relationships between variables. At the same time, the autoregressive paths in the model reflect the temporal stability of variables over time (Talbot et al., 2012). Compared to cross-sectional designs, the cross-lagging model has the following advantages: First, it analyzes time-series data, which better aligns with the logical premise of “temporal precedence” and helps reduce parameter errors that can arise from non-time-series data (Selig and Preacher, 2009). Second, by controlling the autoregressive effect of variables, the model can reduce estimation bias caused by ignoring previous measurements. Third, by comparing the absolute values of cross-lag coefficients, the relative strength of directional longitudinal associations between variables can be further evaluated (McArdle and Nesselroade, 2014). In addition, for data collected using self-report methods, the measurement design with multiple time points also helps to weaken the influence of common method bias and improve the reliability of model results (MacKenzie and Podsakoff, 2012). Based on the above considerations, this study adopts the cross-lagged panel model, aiming to systematically analyze the dynamic interrelationships among psychological resilience, decision confidence, and officiating pressure.

However, it should be noted that the traditional CLPM does not disentangle stable between-person differences from within-person changes over time; therefore, the cross-lagged paths in this study are interpreted as temporal associations rather than definitive causal effects.

## 3 Objects and methods

### 3.1 Objects

This study used convenience sampling to recruit National Class 1 basketball referees, mainly from Sichuan Province and, to a lesser extent, from Shaanxi Province. Eligible participants were those who had officiated in at least three tournament-style basketball events before the study. A longitudinal follow-up investigation was conducted over a period of 6 months, divided into three stages. The follow-up investigations were carried out in July 2025 (T1), October 2025 (T2), and January 2026 (T3). Each wave was separated by a 3-month interval. The data collection period from July 2025 to January 2026 coincided with the university basketball league and the Gongga Cup basketball league. All three waves of data collection were conducted after games rather than before competition. The questionnaire survey was conducted in a combined online and offline format, and a coded identifier derived from the last four digits of each participant’s identification number was used to match responses across the three waves. Invalid questionnaires and participants with incomplete data across any of the three waves were excluded from the final longitudinal analyses. At T1, 447 referees participated in the survey, and 368 referees were ultimately included in the final analysis. The final sample of 368 participants for 35 questionnaire items corresponded to approximately 10.5 participants per item, which meets and slightly exceeds the commonly cited rule-of-thumb of 5–10 participants per item in questionnaire-based research. Nevertheless, this empirical guideline does not replace a formal power analysis (Boateng et al., 2018; DeVellis and Thorpe, 2021). A total of 79 participants were excluded, yielding an attrition rate of 17.67%. We further compared the dropout sample with the retained sample. The results showed no significant differences between participants included in the final analysis and those lost to follow-up in T1 psychological resilience (*t* = 0.116, *p* > 0.05), decision confidence (*t* = −0.331, *p* > 0.05), or officiating pressure (*t* = −0.152, *p* > 0.05), indicating that systematic attrition was unlikely. Among the 368 referees included in the final analysis, 251 were men (68.2%) and 117 were women (31.8%). This gender distribution may reflect the accessibility of the sampled referee group rather than the actual population distribution of collegiate basketball referees in China. Regarding officiating experience, 23.1% had 1–3 years of experience, 56.3% had 3–6 years, and 20.7% had more than 6 years. After obtaining informed consent from the participating referees, three waves of assessment were conducted. The contents and procedures of the three assessments were largely the same, but the order of the questions was adjusted for each wave of assessment. Before each wave of assessment, the referees were informed of the purpose and significance of the study, the meaning of the questionnaire items, the principles of confidentiality, and the importance of independent responses.

This study was approved by the Sports Training College, Chengdu Sport University (Approval No. CTYLL2025008).

### 3.2 Methods

#### 3.2.1 Officiating pressure

The Chinese version of the Officiating and Decision Stress Scale for Referees (SASS-SO), adapted from Anshel’s original scale by Zhang (2020a), was used to assess referees’ perceived stress during officiating (Anshel et al., 2014). It consists of 11 items rated on a 5-point Likert scale (1 = “No stress,” 5 = “Extremely stressful”). The higher the overall score, the greater the overall perceived stress level during the officiating process. In this study, the Cronbach’s *α* coefficients across the three time periods were 0.936, 0.942, and 0.930, respectively. In the present study, the SASS-SO was treated as a single-factor measure of officiating pressure.

#### 3.2.2 Psychological resilience

The Chinese adaptation of the 10-item single-dimensional psychological resilience short form (CD-RISC-10), originally created by Connor and Davidson (2003) and later modified by Yu and Zhang (2007), was used to measure the psychological resilience of the referees. This single-dimensional scale consists of 10 items and uses a 5-point Likert response format (1 = “completely disagree,” 5 = “completely agree”). The higher the total score, the higher the individual’s level of psychological resilience. In this study, the Cronbach’s *α* coefficients of this scale in the three measurements were 0.929 (T1), 0.922 (T2), and 0.942 (T3). In the present study, the CD-RISC-10 was treated as a single-factor measure of psychological resilience.

#### 3.2.3 Decision confidence

In this study, the Chinese-adapted Referee Self-Efficacy Scale (REFS), originally developed by Myers et al. (2012) and later translated and adapted by Zhang (2020b), was used as an operational indicator of referees’ confidence-related efficacy beliefs in officiating. The REFS was originally designed to assess broader referee self-efficacy, including confidence in game knowledge, decision-making, coping with pressure, and communication. Given that confidence in making and maintaining officiating judgments is a central component of referee self-efficacy, the scale was used in the present study as a proxy for decision confidence in the officiating context. The scale consists of 13 items rated on a 5-point Likert scale (1 = “very low confidence,” 5 = “very high confidence”), with higher total scores indicating stronger confidence-related efficacy beliefs in officiating. In this study, the Cronbach’s α coefficients across the three waves were 0.947 (T1), 0.945 (T2), and 0.938 (T3).

### 3.3 Data analysis

After the data were entered into Excel, a coded identifier derived from the last four digits of each participant’s identification number was used solely to facilitate matching across the three waves, and the dataset was then imported into SPSS 27.0 for descriptive and correlational analyses across the three waves. Missing data were handled using listwise deletion; participants with missing data at any wave were excluded from the final longitudinal analyses. Furthermore, confirmatory factor analysis (CFA) was conducted in Amos 29.0 to examine the measurement structure of the study variables. In the present study, psychological resilience, decision confidence, and officiating pressure were modeled as three single-factor latent constructs, each measured by its respective observed items, and tested within a joint measurement model across the three waves. Longitudinal measurement invariance was further examined across T1, T2, and T3, and model fit was evaluated using *χ*^2^/df, CFI, and RMSEA. In addition, composite reliability (CR) and average variance extracted (AVE) were calculated to assess construct reliability and convergent validity.

Amos 29.0 was also used to construct the cross-lagged panel model (CLPM) to examine the longitudinal relationships among psychological resilience, decision confidence, and officiating pressure. To further examine the longitudinal indirect association among the study variables, a three-wave mediation model was specified in which psychological resilience at T1 predicted officiating pressure at T3 through decision confidence at T2. In this model, autoregressive paths were specified from T1 to T2 and from T2 to T3 for psychological resilience, decision confidence, and officiating pressure to account for temporal stability. The indirect effect was tested using the bias-corrected bootstrap approach with 5,000 resamples. Standardized coefficients were reported for the mediation analysis.

## 4 Results

### 4.1 Longitudinal measurement invariance

Before testing the structural model, a joint CFA measurement model including psychological resilience, decision confidence, and officiating pressure was estimated across the three waves. The three latent constructs demonstrated satisfactory measurement properties over time. In addition, composite reliability (CR) and average variance extracted (AVE) were calculated for each construct at each wave. The CR values ranged from 0.922 to 0.947, and the AVE values ranged from 0.540 to 0.622, indicating satisfactory reliability and convergent validity.

The configural invariance model showed a good fit to the data (*χ*^2^/df = 1.507, CFI = 0.967, RMSEA = 0.021). The metric invariance model also demonstrated good fit (*χ*^2^/df = 1.519, CFI = 0.965, RMSEA = 0.022), with only trivial changes in fit indices compared with the configural model (ΔCFI = −0.002, ΔRMSEA = 0.001). The scalar invariance model similarly showed acceptable fit (*χ^2^*/df = 1.566, CFI = 0.961, RMSEA = 0.023), and the changes in fit indices remained within acceptable limits (ΔCFI = −0.006, ΔRMSEA = 0.001). Overall, these findings support longitudinal measurement invariance across T1, T2, and T3, indicating that the three constructs were measured in a sufficiently comparable manner over time and therefore provided an appropriate basis for subsequent longitudinal structural analyses.

### 4.2 Common method bias assessment

To assess the potential influence of common method bias associated with self-report measures, Harman’s single-factor test and several procedural remedies were used. First, well-validated scales that have been widely used in prior domestic and international research were adopted. Then, after presenting the coded information and basic information, all the scale items of psychological resilience, decision confidence, and officiating pressure from time points T1 to T3 were included in the analysis. Using the SPSS software for unrotated factor analysis, the results showed that three time points all extracted three factors with eigenvalues greater than 1. The variance explained by the first factor was 37.77, 36.37, and 35.31%, respectively, all of which were below the critical threshold of 40%. In addition, the use of a three-wave design, coded data matching, confidentiality instructions, and independent responding served as procedural remedies to reduce the potential influence of common method bias. Therefore, the results suggest that severe common method bias was unlikely in the present study.

### 4.3 Correlations among study variables

Table 1 presents the means and standard deviations of psychological resilience, decision confidence, and officiating pressure across the three measurement waves. The table provides an overview of the distribution and variation of the three variables over time.

**Table 1:** Means and standard deviations of psychological resilience, decision confidence, and officiating pressure across T1, T2, and T3.

Variable	T1 M ± SD	T2 M ± SD	T3 M ± SD
Psychological resilience	3.57 ± 1.07	3.68 ± 0.72	2.66 ± 0.76
Decision confidence	2.68 ± 0.97	3.33 ± 0.77	3.10 ± 0.73
Officiating pressure	3.52 ± 0.78	3.74 ± 1.04	2.98 ± 0.73

The correlations among psychological resilience, decision confidence, and officiating pressure across the three waves are presented in Table 2. The three constructs were significantly correlated across the three measurement waves. Among them, psychological resilience was significantly positively correlated with decision confidence and significantly negatively correlated with officiating pressure. Decision confidence was negatively associated with officiating pressure and positively associated with psychological resilience. Officiating pressure was significantly negatively correlated with both psychological resilience and decision confidence.

**Table 2:** Correlation analysis of PR, DC, and OP.

Variables	1	2	3	4	5	6	7	8	9
1 PR T1	1								
2 DC T1	0.508^**^	1							
3 OP T1	−0.415^**^	−0.336^**^	1						
4 PR T2	0.671^**^	0.387^**^	−0.401^**^	1					
5 DC T2	0.472^**^	0.679^**^	−0.362^**^	0.372^**^	1				
6 OP T2	−0.446^**^	−0.414^**^	0.649^**^	−0.399^**^	−0.393^**^	1			
7 PR T3	0.600^**^	0.349^**^	−0.327^**^	0.556^**^	0.308^**^	−0.352^**^	1		
8 DC T3	0.489^**^	0.630^**^	−0.400^**^	0.397^**^	0.643^**^	−0.404^**^	0.365^**^	1	
9 OP T3	−0.439^**^	−0.332^**^	0.614^**^	−0.394^**^	−0.381^**^	0.573^**^	−0.306^**^	−0.433^**^	1

**p < 0.01,***p < 0.001, PR, psychological resilience; DC, decision confidence; OP, officiating pressure.

### 4.4 Cross-lagged analysis of psychological resilience, decision confidence, and officiating pressure

**Figure 1:**
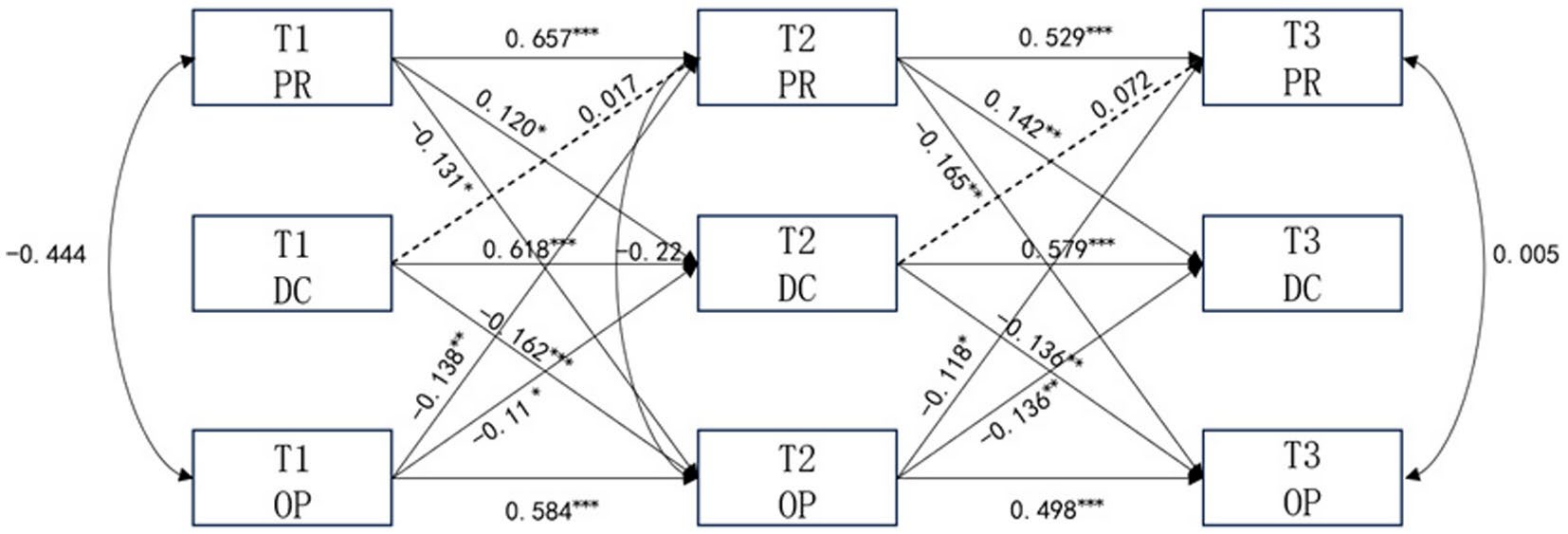
Values are standardized. Solid lines indicate significant paths, and dotted lines indicate non-significant paths. *p < 0.05, **p < 0.01, ***p < 0.001. PR, psychological resilience; DC, decision confidence; OP, officiating pressure.

To examine the longitudinal interrelationships among psychological resilience, decision confidence, and officiating pressure, a cross-lagged panel model was constructed based on the correlation results (Figure 1). Using Amos 29.0 software for maximum likelihood estimation, the hypothesized model demonstrated an acceptable fit. The fitting indices were as follows: *χ*^2^/df = 1.257, CFI = 0.949, TLI = 0.983, RMSEA = 0.026. The autoregressive paths were all moderate to high, indicating substantial temporal stability in psychological resilience, decision confidence, and officiating pressure across the three waves. Specifically, the autoregressive coefficients from T1 to T2 were 0.657, 0.618, and 0.584, respectively, and those from T2 to T3 were 0.529, 0.579, and 0.498. This suggests that the three constructs were relatively stable over time and that the cross-lagged effects should be interpreted after accounting for such stability. The path analysis of the model showed that the psychological resilience at T1 was significantly positively associated with the decision confidence at T2 (*β* = 0.12, *p* < 0.05), and the psychological resilience at T2 was significantly positively associated with the decision confidence at T3 (*β* = 0.142, *p* < 0.01); the decision confidence at T1 was significantly negatively associated with the officiating pressure at T2 (*β* = −0.162, *p* < 0.001), but it was not significantly associated with the psychological resilience at T2 (*β* = 0.017, *p* > 0.05); the decision confidence at T2 was not significantly associated with the psychological resilience at T3 (*β* = 0.072, *p* > 0.05), but it was significantly negatively associated with the officiating pressure at T3 (*β* = −0.136, *p* < 0.01); the officiating pressure at T1 was significantly negatively associated with the psychological resilience at T2 (*β* = −0.138, *p* < 0.01) and the decision confidence at T2 (*β* = −0.11, *p* < 0.05), and the officiating pressure at T2 was significantly negatively associated with the psychological resilience at T3 (*β* = −0.118, *p* < 0.05) and the decision confidence at T3 (*β* = −0.136, *p* < 0.01).

### 4.5 Analysis of the mediating effect of decision confidence

The indirect effect analysis focused on the path from T1 psychological resilience to T3 officiating pressure via T2 decision confidence because this pathway directly corresponded to the theory-driven hypothesis of the present study.

In the longitudinal mediation model, autoregressive paths for psychological resilience, decision confidence, and officiating pressure across adjacent waves were included to account for temporal stability. To examine whether decision confidence was involved in an indirect longitudinal association between psychological resilience and officiating pressure, the indirect effect of the hypothesized pathway from T1 psychological resilience to T3 officiating pressure through T2 decision confidence was tested using the non-parametric bias-corrected percentile bootstrap method. Standardized coefficients were reported in the mediation analysis, and the total effect was defined as the sum of the direct effect and the indirect effect. The results are presented in Table 3. The bootstrap mediation analysis showed that the direct effect of the path from T1 psychological resilience to T3 officiating pressure was −0.347, with a 95% confidence interval of [−0.454, −0.231], which did not include zero, indicating a significant direct effect. For the pathway T1 psychological resilience → T2 decision confidence → T3 officiating pressure, the indirect effect was −0.117, with a 95% confidence interval of [−0.180, −0.060], which also excluded zero, indicating a significant indirect effect. The indirect effect accounted for 25.2% of the total effect, suggesting that decision confidence was involved in a partial indirect longitudinal association between psychological resilience and officiating pressure.

**Table 3:** Test of mediating effect.

Mediating path	Effect size	SE	95%CI		*p*
			Lower	Upper	
PR T1 → DC T2 → OP T3 (indirect effect)	−0.117	0.030	−0.180	−0.060	<0.001
PR T1 → OP T3 (direct effect)	−0.347	0.057	−0.454	−0.231	<0.001
PR T1 → OP T3 (total effect)	−0.464	0.045	−0.548	−0.371	<0.001

All coefficients are standardized estimates. Total effect = direct effect + indirect effect.

## 5 Discussion

To investigate the longitudinal relationships among psychological resilience, decision confidence, and officiating pressure, this study conducted three follow-up surveys of college basketball referees using a questionnaire-based approach. The results indicated significant concurrent and longitudinal associations among the three variables.

### 5.1 The relationship between psychological resilience, decision confidence, and officiating pressure

First, the findings indicate that psychological resilience was negatively associated with subsequent officiating pressure over time and was positively associated with subsequent decision confidence. The cross-lagged analysis suggests the directional temporal association of psychological resilience with pressure and decision confidence through temporal control. This result is consistent with previous studies. Previous research has shown that if referees have good psychological resilience, it can help them actively adapt to adversity and pressure in the game, thereby helping referees improve their performance and professional skills (Liu, 2016). At the same time, psychological resilience can be regarded as the “psychological defense system” of referees to cope with pressure. It may not only be associated with lower perceived officiating pressure but also may be associated with higher referees’ ability to resist pressure through intermediary mechanisms such as improving self-efficacy in officiating, ultimately improving the ability to maintain stable judgments in on-court officiating (Guillén and Feltz, 2011; Anshel et al., 2014). This may be particularly important for college basketball referees. Further research shows that psychological resilience does not simply counteract pressure, but as a core psychological resource, it can reduce the negative impact of pressure through multiple paths and convert it into positive effects (Fletcher and Sarkar, 2013). Moreover, it may help referees remain focused under pressure and maintain confidence in their judgments. More importantly, psychological resilience gives referees a strong recovery ability and learning ability, enabling them to recover quickly from on-court mistakes and transform the pressure-induced experiences into personal growth motivation (Sarkar and Fletcher, 2014; Secades et al., 2016). Therefore, psychological resilience is a key component of the overall psychological makeup of excellent basketball referees. Additionally, research shows that referees with higher levels of psychological resilience will exhibit higher decision confidence in the next time period. It has been shown that psychological resilience is related to decision confidence, and psychological resilience is also a protective factor for decision confidence. Individuals with high psychological resilience often have higher decision confidence (Fletcher and Sarkar, 2012; Gucciardi et al., 2017). As the core psychological resource of referees, psychological resilience provides important foundational guarantees for decision confidence by controlling emotions, improving cognition, and enhancing the ability to resist risks. It enables referees to make rational judgments and make decisive decisions in high-pressure environments (Guo and Liu, 2024), which is crucial for ensuring the accuracy of referees’ judgments in on-court situations. In practice, various pressures often occur on the court, sometimes due to referees’ incorrect or missed judgments, causing dissatisfaction among athletes and spectators. However, sometimes even normal judgments may result in impolite behaviors due to the psychological tendencies of the audience. Therefore, pressure exists all the time, but the ability to resist pressure and decision confidence can be systematically cultivated. Through training, referees can maintain rational judgments and make decisive decisions in high-pressure environments.

Second, according to the cross-lagged panel model, officiating pressure was negatively associated with subsequent decision confidence and psychological resilience over time. In other words, referees with higher officiating pressure tended to report lower psychological resilience and decision confidence at the subsequent wave. These findings support the theoretical framework proposed earlier. COR theory suggests that prolonged officiating pressure may consume psychological resources, while cognitive appraisal theory indicates that pressure responses depend on how referees evaluate situational demands relative to their coping capacity. Psychological resilience is the psychological reserve resource that an individual uses to cope with stress and restore balance; decision confidence is the cognitive and judgment resource that referees use to make decisive decisions in the rapidly changing game situation. Both belong to the key individual resources in the resource conservation theory (Hobfoll, 2001). This study shows that when referees are continuously exposed to high-pressure situations during the game, their psychological resources (including attention, psychological resilience, and self-efficacy) will be continuously depleted, making it difficult for them to maintain high levels of psychological resilience and decision confidence. This is consistent with previous studies on “work pressure eroding psychological capital” (Hobfoll, 2001; Kittel et al., 2025). Therefore, when referees are under multiple pressures such as game intensity, coach pressure, and audience dissatisfaction during the game, they may have problems such as questioning their own judgments, being afraid to communicate with players, and reducing their game management skills, thereby reducing their officiating level and accuracy. This implies that greater focus should be placed on protecting and restoring referees’ psychological resources to avoid resource depletion due to long-term participation in high-pressure officiating competitions and emotional avoidance, which can lead to a decline in officiating ability and officiating burnout.

Third, the cross-lagged model showed that decision confidence has a significant negative longitudinal association with college basketball referees’ officiating pressure but does not predict subsequent psychological resilience. This implies that referees with higher decision confidence will experience lower officiating pressure in the next period. This finding is broadly consistent with previous research. High decision confidence helps referees generate self-confidence and a sense of hope, and a positive sense of hope helps referees develop psychological resilience to resist pressure (Locke, 1997; Li and Song, 2012). This internal certainty can be transformed into decisive judgments and self-confidence during officiating, effectively cushioning psychological pressure stemming from external sources (such as coach pressure, spectator noise, and high-stakes decisions). On the contrary, referees with insufficient decision confidence are more likely to experience self-doubt and anxiety due to the uncertainty of the situation during officiating, thereby feeling stronger officiating pressure and affecting their on-the-spot judgments and decisions (Nevill et al., 2002). The main responsibility of referees is to objectively reflect the situation on the court, but mistakes and missed calls are inevitable. However, if referees get stuck in emotional turmoil due to a single incorrect decision and even try to “make up” or “balance” the previous mistake through subsequent decisions, it will not only lead to further judgment errors but also weaken their decision confidence, thereby damaging the confidence and credibility of the referees’ officiating (García-Santos et al., 2020). On the other hand, decision confidence cannot predict psychological resilience, possibly due to the different fundamental attributes of the two. Psychological resilience is a stable personal resource that requires long-term accumulation. On the contrary, decision confidence is a situational resource influenced by on-the-spot performance, with significant variations, mainly regulating immediate pressure, and is difficult to form into a stable psychological trait in the short term. This indicates that in the training of referees, we should avoid overly relying on short-term confidence enhancement and instead focus more on the systematic cultivation of psychological resilience (Jones et al., 2002).

Finally, in addition to the aforementioned direct temporal prediction relationship, this study, through mediation effect analysis, found that decision confidence plays a mediating role in the relationship between psychological resilience and officiating pressure. Specifically, at the T1 time point, psychological resilience can enhance decision confidence at the T2 time point, thereby indirectly reducing the pressure at the T3 time point. At the same time, psychological resilience at the T1 time point also directly affects the pressure at the T3 time point. This finding suggests a possible longitudinal pathway through which psychological resilience may be linked to later officiating pressure: Psychological resilience not only directly acts as a stress-buffering trait to mitigate on-court pressure but also may be indirectly associated with lower later officiating pressure through higher intervening decision confidence. This finding supports and deepens the previous theory that psychological resources influence the outcome of stress through the cognitive assessment pathway (Benight and Bandura, 2004; Guo and Liu, 2024). At the practical level, it indicates that for the psychological intervention of college student referees, if both their psychological resilience and decision confidence are enhanced simultaneously, it can have a synergistic effect, more effectively helping college student referees cope with the pressure of officiating and thereby potentially helping them better cope with officiating pressure and supporting more stable on-site functioning.

## 6 Implications and recommendations

These research results are important for enhancing the psychological stability and on-court performance of college basketball referees. The research indicates that there is a mutual and dynamic relationship among psychological resilience, decision confidence, and pressure during officiating. A systematic training system integrating psychological resilience and decision confidence can be established. In daily training, high-pressure situation simulations and post-game reviews should be incorporated to help referees continuously accumulate psychological capital during skill improvement. In practice, stratified psychological training should be implemented: for novice referees, the focus should be on pressure management, attention regulation during officiating, and post-officiating emotional counseling; for referees with more experience, emphasize the improvement of pressure cognition, enabling them to transform challenges into growth opportunities and enhancing their psychological resilience and recovery ability in critical judgments (Weinberg and Williams, 2001; Hays et al., 2009). At the same time, the evaluation of referees should be optimized, including dimensions such as psychological stability and decision quality under high pressure (Mascarenhas et al., 2005). Psychological resources can be continuously accumulated through pre-game meetings and pre-game communication, creating a favorable officiating atmosphere, and conducting psychological counseling after the game (MacMahon et al., 2014).

## 7 Limitations and prospects

Although this study longitudinally examined the relationships among psychological resilience, decision confidence, and officiating pressure in college basketball referees, several limitations should be acknowledged. First, the traditional CLPM does not distinguish between stable between-person differences and within-person changes over time; therefore, the findings should be interpreted as predictive associations rather than causal effects. Second, the sample was limited to National Class 1 college basketball referees, mainly from Sichuan Province and partly from Shaanxi Province, which may limit the generalizability of the findings. Third, all variables were assessed using self-report measures, and the REFS was originally developed to assess broader referee self-efficacy rather than decision confidence alone. Finally, only three waves of data were collected, which may have limited the precision with which dynamic processes were captured.

## 8 Conclusion

This study, through a longitudinal survey of 368 college basketball referees, found that (1) psychological resilience was positively associated with decision confidence over time and was negatively associated with officiating pressure; (2) decision confidence was negatively associated with officiating pressure over time; (3) officiating pressure was significantly negatively associated with subsequent psychological resilience and decision confidence; and (4) decision confidence mediated the indirect longitudinal relationship between psychological resilience and officiating pressure. In summary, there are complex, dynamic interrelationships among psychological resilience, decision confidence, and officiating pressure. Enhancing psychological resilience and fostering decision confidence may represent important psychological strategies to help college basketball referees better cope with officiating pressure and maintain more stable officiating performance.

## Data Availability

The raw data supporting the conclusions of this article will be made available by the authors, without undue reservation.

## References

[ref1] Aguilar J. L. Castillo-Rodriguez A. Chinchilla-Minguee J. L. Onetti-Onetti W. (2021). Relationship between age, category and experience with the soccer referee's self-efficacy. Peerj 9:e11472. doi: 10.7717/peerj.11472 34178441 PMC8199919

[ref2] Anshel M. H. Sutarso T. Ekmekci R. Saraswati I. W. (2014). A model linking sources of stress to approach and avoidance coping styles of Turkish basketball referees. J. Sports Sci. 32, 116–128. doi: 10.1080/02640414.2013.816762, 24015999

[ref3] Aspinwall L. G. Taylor S. E. (1997). A stitch in time: self-regulation and proactive coping. Psychol. Bull. 121, 417–436. doi: 10.1037/0033-2909.121.3.417, 9136643

[ref4] Benight C. C. Bandura A. (2004). Social cognitive theory of posttraumatic recovery: the role of perceived self-efficacy. Behav. Res. Ther. 42, 1129–1148. doi: 10.1016/j.brat.2003.08.008, 15350854

[ref5] Boateng G. O. Neilands T. B. Frongillo E. A. Melgar-Quiñonez H. R. Young S. L. (2018). Best practices for developing and validating scales for health, social, and behavioral research: a primer. Front. Public Health 6:149. doi: 10.3389/fpubh.2018.00149, 29942800 PMC6004510

[ref6] Connaughton D. Wadey R. Hanton S. Jones G. (2008). The development and maintenance of mental toughness: perceptions of elite performers. J. Sports Sci. 26, 83–95. doi: 10.1080/02640410701310958, 17852671

[ref7] Connor K. M. Davidson J. R. (2003). Development of a new resilience scale: the Connor-Davidson resilience scale (CD-RISC). Depress. Anxiety 18, 76–82. doi: 10.1002/da.10113, 12964174

[ref8] Coulter T. J. Mallett C. J. Gucciardi D. F. (2010). Understanding mental toughness in Australian soccer: perceptions of players, parents, and coaches. J. Sports Sci. 28, 699–716. doi: 10.1080/02640411003734085, 20496223

[ref9] DeVellis R. F. Thorpe C. T. (2021). Scale Development: Theory and Applications. Thousand Oaks, California (USA): Sage Publications.

[ref10] Diotaiuti P. Falese L. Mancone S. Purromuto F. (2017). A structural model of self-efficacy in handball referees. Front. Psychol. 8:811. doi: 10.3389/fpsyg.2017.00811, 28572783 PMC5435812

[ref11] Fletcher D. Sarkar M. (2012). A grounded theory of psychological resilience in Olympic champions. Psychol. Sport Exerc. 13, 669–678. doi: 10.1016/j.psychsport.2012.04.007

[ref12] Fletcher D. Sarkar M. (2013). Psychological resilience a review and critique of definitions, concepts, and theory. Eur. Psychol. 18, 12–23. doi: 10.1027/1016-9040/a000124

[ref13] Gao F. Chen Y. Liu P. Jiang Z. Huang X. (2017). An analysis of the predictive power of psychological resilience, loneliness, and self-efficacy on the subjective well-being of the elderly. Psychol. Behav. Res. 15, 227–232+239. doi: 10.3969/j.issn.1672-0628.2017.02.013

[ref14] García-Santos D. Gómez-Ruano M. Vaquera A. Ibáñez S. (2020). Systematic review of basketball referees’ performances. Int. J. Perform. Anal. Sport 20, 495–533. doi: 10.1080/24748668.2020.1758437

[ref15] Gross J. J. John O. P. (2003). Individual differences in two emotion regulation processes: implications for affect, relationships, and well-being. J. Pers. Soc. Psychol. 85, 348–362. doi: 10.1037/0022-3514.85.2.348, 12916575

[ref16] Gucciardi D. F. Hanton S. Fleming S. (2017). Are mental toughness and mental health contradictory concepts in elite sport? A narrative review of theory and evidence. J. Sci. Med. Sport 20, 307–311. doi: 10.1016/j.jsams.2016.08.006, 27568074

[ref17] Guillén F. Feltz D. L. (2011). A conceptual model of referee efficacy. Front. Psychol. 2:25. doi: 10.3389/fpsyg.2011.00025, 21713174 PMC3111226

[ref18] Guo Y. Liu Y. (2024). The influence of martial arts practice experience on the risk of bullying among junior high school students: the chain-like mediating role of psychological resilience and self-efficacy. Sports Sci. 45, 85–93. doi: 10.13598/j.issn1004-4590.2024.05.009

[ref19] Hays K. Thomas O. Maynard I. Bawden M. (2009). The role of confidence in world-class sport performance. J. Sports Sci. 27, 1185–1199. doi: 10.1080/02640410903089798, 19724964

[ref20] Hill D. M. Hemmings B. (2015). A phenomenological exploration of coping responses associated with choking in sport. Qual. Res. Sport Exerc. Health 7, 521–538. doi: 10.1080/2159676x.2014.981573

[ref21] Hobfoll S. E. (2001). The influence of culture, community, and the nested-self in the stress process: advancing conservation of resources theory. Appl. Psychol. 50, 337–421. doi: 10.1111/1464-0597.00062

[ref22] Jones G. Hanton S. Connaughton D. (2002). What is this thing called mental toughness? An investigation of elite sport performers. J. Appl. Sport Psychol. 14, 205–218. doi: 10.1080/10413200290103509

[ref23] Kabat-Zinn J. (2003). Mindfulness-based interventions in context: past, present, and future. Clin. Psychol. Sci. Pract. 10, 144–156. doi: 10.1093/clipsy.bpg016

[ref24] Kittel A. Lindsay R. Larkin P. Spittle M. Cunningham I. (2025). The effectiveness of decision-making training in team-sport officials: a systematic review and meta-analysis. Psychol. Sport Exerc. 79:102841. doi: 10.1016/j.psychsport.2025.102841, 40107585

[ref25] Lazarus R. S. Folkman S. (1984). Stress, Appraisal, and Coping. New York, NY (USA): Springer Publishing Company. doi: 10.1016/0005-7967(85)90087-7

[ref26] Leicht A. Gomez M. Woods C. (2017). Team performance indicators explain outcome during women’s basketball matches at the Olympic games 5, 96. doi: 10.3390/sports5040096 PMC5969024 29910456

[ref27] Li J. Song H. (2012). The relationship between the stress evaluation results of athletes and their coping styles, as well as the moderating effect of general self-efficacy. Zhejiang Sports Sci. 34, 99–102. doi: 10.3969/j.issn.1004-3624.2012.01.027

[ref28] Liu Z. B. (2016). Research on the Training System for High-Level Basketball Referees in China. China University of Mining and Technology.

[ref29] Liu L. Wu D. Wang L. L. Qu Y. T. Wu H. (2020). Effort-reward imbalance, resilience and perceived organizational support: a moderated mediation model of fatigue in Chinese nurses. Risk Manag. Healthc. Policy 13, 893–901. doi: 10.2147/rmhp.S259339, 32801964 PMC7394598

[ref30] Locke E. A. (1997). Self-efficacy: the exercise of control. Pers. Psychol. 50:801.

[ref31] MacKenzie S. B. Podsakoff P. M. (2012). Common method bias in marketing: causes, mechanisms, and procedural remedies. J. Retail. 88, 542–555. doi: 10.1016/j.jretai.2012.08.001

[ref32] MacMahon C. Mascarenhas D. Plessner H. Pizzera A. Oudejans R. Raab M. (2014). Sports Officials and Officiating: Science and Practice. London, UK: Routledge.

[ref33] Mascarenhas D. R. Collins D. Mortimer P. (2005). Elite refereeing performance: developing a model for sport science support. Sport Psychol. 19, 364–379. doi: 10.1123/tsp.19.4.364

[ref34] McArdle J. J. Nesselroade J. R. (2014). Longitudinal Data Analysis using Structural Equation Models. Washington, DC (USA): American Psychological Association.

[ref35] McManama O’Brien K. H. Rowan M. Willoughby K. Griffith K. Christino M. A. (2021). Psychological resilience in young female athletes. Int. J. Environ. Res. Public Health 18:8668. doi: 10.3390/ijerph18168668, 34444426 PMC8392459

[ref36] Mellalieu S. D. Neil R. Hanton S. Fletcher D. (2009). Competition stress in sport performers: stressors experienced in the competition environment. J. Sports Sci. 27, 729–744. doi: 10.1080/02640410902889834, 19424897

[ref37] Myers N. D. Feltz D. L. Guillén F. Dithurbide L. (2012). Development of, and initial validity evidence for, the referee self-efficacy scale: a multistudy report. J. Sport Exerc. Psychol. 34, 737–765. doi: 10.1123/jsep.34.6.737, 23204357

[ref38] Nevill A. M. Balmer N. J. Williams A. M. (2002). The influence of crowd noise and experience upon refereeing decisions in football. Psychol. Sport Exerc. 3, 261–272. doi: 10.1016/s1469-0292(01)00033-4

[ref39] Rogosa D. (1980). Comparing nonparallel regression lines. Psychol. Bull. 88:307. doi: 10.1037/0033-2909.88.2.307

[ref40] Sabag E. Lidor R. Arnon M. Morgulev E. Bar-Eli M. (2023). Teamwork and decision making among basketball referees: the 3PO principle, refereeing level, and experience. J. Hum. Kinet. 89:313. doi: 10.5114/jhk/169439, 38053959 PMC10694708

[ref41] Samuel R. D. Tenenbaum G. Galily Y. (2021). An integrated conceptual framework of decision-making in soccer refereeing. Int. J. Sport Exerc. Psychol. 19, 738–760. doi: 10.1080/1612197X.2020.1766539

[ref42] Sarkar M. Fletcher D. (2014). Psychological resilience in sport performers: a review of stressors and protective factors. J. Sports Sci. 32, 1419–1434. doi: 10.1080/02640414.2014.901551, 24716648

[ref43] Schuurman N. K. Ferrer E. de Boer-Sonnenschein M. Hamaker E. L. (2016). How to compare cross-lagged associations in a multilevel autoregressive model. Psychol. Methods 21:206. doi: 10.1037/met0000062, 27045851

[ref44] Secades X. G. Molinero O. Salguero A. Barquín R. R. de la Vega R. Márquez S. (2016). Relationship between resilience and coping strategies in competitive sport. Percept. Mot. Skills 122, 336–349. doi: 10.1177/0031512516631056, 27420325

[ref45] Selig J. P. Preacher K. J. (2009). Mediation models for longitudinal data in developmental research. Res. Hum. Dev. 6, 144–164. doi: 10.1080/15427600902911247

[ref46] Southwick S. M. Bonanno G. A. Masten A. S. Panter-Brick C. Yehuda R. (2014). Resilience definitions, theory, and challenges: interdisciplinary perspectives. Eur. J. Psychotraumatol. 5:25338. doi: 10.3402/ejpt.v5.25338, 25317257 PMC4185134

[ref47] Talbot L. S. Stone S. Gruber J. Hairston I. S. Eidelman P. Harvey A. G. (2012). A test of the bidirectional association between sleep and mood in bipolar disorder and insomnia. J. Abnorm. Psychol. 121, 39–50. doi: 10.1037/a0024946, 21842957 PMC3477806

[ref48] Wang X. (2016). Research on the Psychological Stress of Tennis Referees and Their Coping Strategies. Kunming, China: Yunnan Normal University.

[ref49] Wang C. (2020). Research on the Psychological Stress and Coping Strategies of Basketball Referees during On-Site Enforcement in Shandong Province. Jinan, China: Shandong Normal University.

[ref50] Wang Q. Yang P. (2021). The relationship between self-control and emotional stability of high-level basketball referees in China: the chain mediating role of mood state and psychological resilience. Sports Sci. 42, 86–95. doi: 10.13598/j.issn1004-4590.2021.06.013

[ref51] Weinberg R. Williams J. (2001). “Integrating and implementing a psychological skills training program,” in Applied Sport Psychology: Personal Growth to Peak Performance, ed. J. M. Williams. vol. 4 (Mountain View, CA: Mayfield Publishing Company), 347–377.

[ref52] Wu M. Yan H. (2020). Analysis of the pressure on basketball referees in minority areas and countermeasures - taking Liangshan Yi autonomous prefecture as an example. China Sport Sci. Technol. 41, 44–45+47. doi: 10.14038/j.cnki.tykj.2020.05.018

[ref53] Xi J. Z. Sang B. (2008). A review and prospect of resilience research. Psychol. Sci. 4, 995–998+977. doi: 10.16719/j.cnki.1671-6981.2008.04.044

[ref54] Yu X. Zhang J. (2007). Application comparison of self-resilience scale and Connor-Davidson resilience scale. Psychol. Sci. 5, 1169–1171. doi: 10.16719/j.cnki.1671-6981.2007.05.035

[ref55] Zhang Q. (2020a). The impact of stressors on the satisfaction of football referees' decisions: the chain mediating role of positive coping styles and coping efficacy. Hubei Sports Technol. 39, 891–895.

[ref56] Zhang Q. (2020b). The mediating effect of self-efficacy between job burnout and decision satisfaction of football referees. Sports Res. Educ. 35, 39–44. doi: 10.16207/j.cnki.2095-235x.2020.04.007

[ref57] Zheng S. Muratli B. (2025). The relationship between self-efficacy and fatigue of high-level wrestling judges in China: the complete mediating role of psychological resilience. Chinese J. Health Psychol. 33, 771–775. doi: 10.13342/j.cnki.cjhp.2025.05.026

